# Effects of 8 weeks of resistance training in combination with a high protein diet on body composition, muscular performance, and markers of liver and kidney function in untrained older ex-military men

**DOI:** 10.3389/fnut.2023.1205310

**Published:** 2023-06-29

**Authors:** Reza Bagheri, Abolfazl Shakibaee, Donny M. Camera, Vahid Sobhani, Hamid Ghobadi, Eisa Nazar, Hadi Fakhari, Fred Dutheil

**Affiliations:** ^1^Exercise Physiology Research Center, Lifestyle Institute, Baqiyatallah University of Medical Sciences, Tehran, Iran; ^2^Department of Health and Biostatistics, Swinburne University, Melbourne, VIC, Australia; ^3^Department of Exercise Physiology, Ferdowsi University of Mashhad, Mashhad, Iran; ^4^Psychiatry and Behavioral Sciences Research Center, Addiction Institute, Mazandaran University of Medical Sciences, Sari, Iran; ^5^Department of Exercise Physiology, University of Isfahan, Isfahan, Iran; ^6^CNRS, LaPSCo, Physiological and Psychosocial Stress, CHU Clermont-Ferrand, University Hospital of Clermont-Ferrand, Preventive and Occupational Medicine, Université Clermont Auvergne, Clermont-Ferrand, France

**Keywords:** resistance exercise, skeletal muscle adaptation, nutrition, aging, protein

## Abstract

**Background:**

The effects of a high protein diet in combination with chronic resistance training (RT) on skeletal muscle adaptation responses in untrained older ex-military men is unknown. Therefore, we compared the effects of 8 weeks of RT in combination with either a high (1.6 g/kg/d) or low protein diet (0.8 g/kg/d) on body composition [skeletal muscle mass (SMM) and body fat percentage (BFP)], muscular strength, power, and endurance (upper and lower body), markers of liver [alanine transaminase (ALT), aspartate aminotransferase (AST), and gamma-glutamyl transferase (GGT)] and kidney (creatinine and urea) function, and lipid profile low-density lipoprotein (LDL), high-density lipoprotein (HDL), and cholesterol levels in a cohort of healthy, untrained older ex-military males.

**Methods:**

Forty healthy untrained older ex-military males (age: 61 ± 2 yr, body mass index: 23.2 ± 1.3 kg.m^−2^) performed 8 weeks (three sessions·w^−1^) of RT with either 1.6 g/kg/d (RHP; *n* = 20) or 0.8 g/kg/d of protein (RLP; *n* = 20). Body composition (assessed by Inbody 720), muscular strength (1-RM for chest and leg press), power (Wingate test), endurance (75% 1-RM for chest and leg press), and markers of liver and kidney function (biochemical kits) were assessed pre and post-intervention.

**Results:**

SMM and muscular strength (upper and lower body) increased post-intervention in both groups and were significantly greater in RHP compared to RLP, while muscular power increased to the same extent in both groups (*p* < 0.05) with no between-group differences (*p* > 0.05). In contrast, there were no post-intervention changes in muscular endurance, HDL, and BFP remained in either group (*p* > 0.05). ALT and creatinine significantly increased in RHP compared to RLP while GGT, AST, and urea only increased in the RLP group (*p* < 0.05). LDL and cholesterol significantly decreased in both groups (*p* < 0.05).

**Conclusion:**

A daily intake of 1.6 g/kg/d protein was superior to 0.8 g/kg/d (current recommended daily intake) for promoting greater improvements in SMM and muscle strength and thus may be a more suitable level of intake for promoting such adaptive responses. Notwithstanding observed between-group differences in ALT and creatinine and the fact that levels remained within normal ranges, it is feasible to conclude that this daily protein intake is efficacious and well tolerated by healthy, untrained older ex-military males.

## Introduction

Quality and quantity of skeletal muscle and physical function are of importance with advancing age (1). Indeed, skeletal muscle mass (SMM) and strength/power decline at rates of 0.8–1% and 2–3% each year, respectively (2). While it is difficult to precisely pinpoint the prevalence of sarcopenia in the community, current statistics indicate that 2.5 to 30% of older persons (≥ 60 years) are classified as having low SMM (3, 4). The delicate balance between muscle protein synthesis and muscle protein breakdown determines SMM (5). The existence of anabolic resistance reduces the sensitivity of older adults to anabolic stimuli and dietary protein intake, irrespective of the fact that evidence suggests that skeletal muscle is typically well-maintained in young, healthy people who do not engage in scheduled physical activity (6). Preserving SMM in the older population is thus complicated. Consequently, the selection and implementation of effective physical activity and dietary approaches to combat the reduced anabolism in the skeletal muscle of older persons may assist to enhance muscular hypertrophy or at the very least aid in the maintenance of SMM (7), particularly in retired military personnel.

Resistance training (RT) serves as the most efficient non-pharmaceutical stimulant for increasing SMM, strength, and physical function, and is consequently the most effective intervention for age-related sarcopenia (8–11). Although muscle protein synthesis is significantly enhanced after RT is executed in a post-adaptive state (i.e., when fasting), muscle protein breakdown is also elevated, and the muscle net protein balance consequently maintains negative (12). Protein intake, especially essential amino acids in somewhat close temporal approximation, such as <3–4 h after RT, increases muscle protein synthesis while decreasing muscle protein breakdown, which supports a favorable muscle net protein balance (13). Regarding total daily protein intake, the current recommended daily allowance has remained stable over many decades at 0.8 g/kg/d (14). Due to the prevalence of anabolic resistance in older adults, certain expert groups (15, 16) have advised protein needs between 1 and 1.5 g/kg/d (16). In support, higher daily protein intakes (>78.5 g/d) have been linked to increased SMM retention in older males (17). In addition, a large number of research on older persons indicate that increased protein intake is favorably linked with step count and adversely associated with inactive time in older adults (18). Additionally, older persons who consumed over 1 g/kg/d protein had a 22% lower risk of functional impairment as measured by 19 functional tests (19) while a six-year study in persons aged 60 and older observed a correlation between protein intake and grip strength maintenance (20). While these findings collectively suggest that older persons who desire to retain SMM and function may get substantial advantages from a protein intake in excess of the recommended daily allowance, several lines of evidence suggest a higher protein diet is hazardous to renal and bone health (21–24). However, these perceptions remain unsupported (14, 25–29). In the absence of renal disease, greater protein intake is linked with normal kidney function (28) and is associated with an enhanced glomerular filtration rate (25). Furthermore, protein intake over the recommended daily allowance may be advantageous for bone health (14) and may assist to lower the risk of hip fracture and bone mineral density loss (26, 29). Therefore, despite the fact that the daily protein intake of the majority of older individuals is insufficient and worsened with age, it is safe for older adults to consume protein over the recommended daily allowance (~1.6 g/kg/d), and this seems to be advantageous for combating sarcopenia.

Military recruit training programs are intended to transform untrained civilians into trained soldiers; consequently, physical training is necessarily rigorous, consisting of a combination of endurance training, strength and conditioning, obstacle courses, swimming, circuit training, and loaded marching (30). Although numerous studies (both original and review) to date have been published in older adults following RT and protein (dietary or supplemental form) intake indicating improved body composition and muscular performance (8, 31, 32), research on retired military older adults is lacking. Therefore, we compared the effects of 8 weeks of RT in the combination of 1.6 g/kg/d of protein vs. 0.8 g/kg/d on body composition, muscular performance (strength, power, and endurance), and biochemical markers of liver and kidney function in a cohort of healthy, untrained older ex-military males.

## Methods

### Participants

The current research included 40 healthy, untrained ex-military older males (>55 years). At the gym, interested participants were educated about the research and testing procedures in person. Participants were required to self-report a health and fitness history questionnaire, confirming that they had not exercised in the previous 6 months, sleeping for at least seven to 8 h per day, not taking any steroids or illegal agents known to increase SMM in the previous year, less than 0.8 g/kg/d of protein ingestion, and being free of musculoskeletal disorders. Participants who were deemed eligible based on the aforementioned criteria supplied written and verbal agreement to participate. In addition, while agreeing, a medical history questionnaire was gathered, and individuals were requested to return to complete the research procedures. Participants were ex-military for five to 10 years since they retired and were in the military for 30 to 35 years. The protocol was reviewed by the Institutional Human Subject Committee and the Ethics Committee of Baqiyatallah University of Medical Sciences (IR.BMSU.BAQ.REC.1401.132) and carried out in accordance with the Declaration of Helsinki. This study has been registered with the Iranian Registry of Clinical Trials (IRCT20191204045612N3).

### Study design

**Figure 1:**
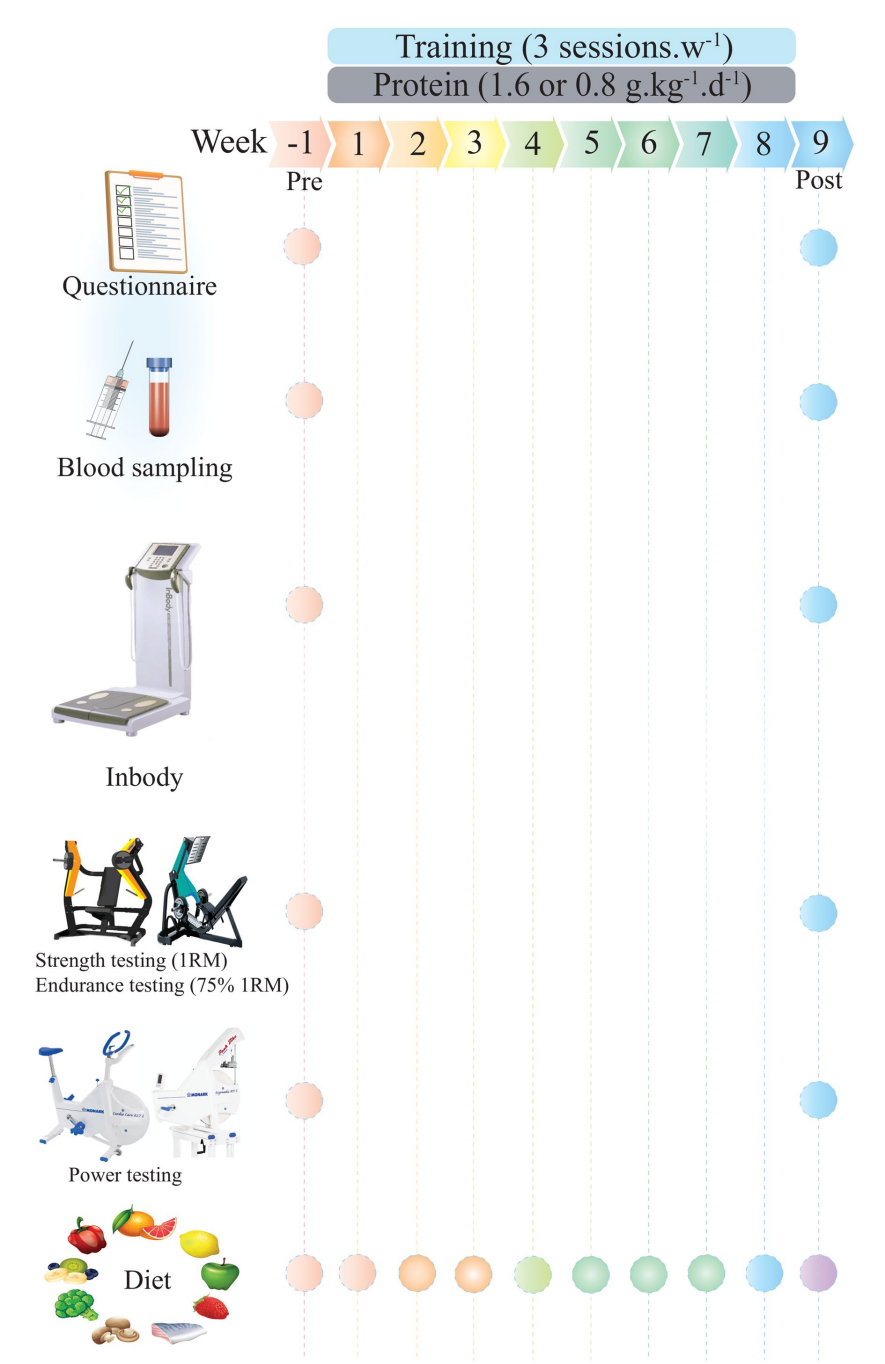
Overview of the study timeline.

Figure 1 depicts an overview of the study protocol. Following baseline measures (detailed below), participants were acquainted with the study tests and procedures before being randomly assigned (using a digital tool)^1^ to one of two groups for an 8-week intervention involving either RT + 1.6 g/kg/d of protein (RHP; *n* = 20) or RT + 0.8 g/kg/d of protein (RLP; *n* = 20). Measurements were taken at the same time of day at baseline and week 9 (the week after the training protocols were finished). Participants first completed four preliminary testing days: questionnaires were assessed on the first visit; blood sampling and body composition were performed on the second visit; muscular strength and endurance (75% 1-RM) were performed on the third visit; and a muscular power test was performed on the fourth visit. Following the completion of these tests, participants met with the study nutritionist for an initial session to establish dietary preferences as well as target protein and energy intakes before beginning training procedures. The Pittsburgh Sleep Quality Index (PSQI) and the General Health Questionnaire-28 (GHQ-28) were used to measure sleep quality and health status, respectively. All tests were carried out in the precise sequence specified for all time measurements.

### Anthropometry and body composition

Participants were instructed to arrive at the lab hydrated following an overnight fast, and a 24-h food recall was gathered before testing. To avoid hydration status mistakes, participants were urged to urinate entirely within 30 min of the test and to abstain from caffeinated drinks, alcohol, and other diuretics 12 h before measurements. Body mass and height were measured using a digital scale (Lumbar, China) to the closest 0.1 kg and a stadiometer (Race industrialization, China), respectively. As previously described, a multi-frequency bioelectrical impedance device (Inbody 720, South Korea) was used to measure SMM, body fat percentage (BFP), and body mass index (BMI) (9). Before the measurement, the participant’s palms and soles were cleaned with an electrolyte tissue. Subjects then stood on the InBody 720, placing the soles of their feet on the electrodes. The instrument derived the participant’s body mass, and their age and sex were manually entered into the display by a researcher. Subjects then grasped the handles of the unit ensuring that the palm and fingers of each hand made direct contact with the electrodes, with arms fully extended and abducted approximately 20^o^. Analysis of body composition was determined by the unit with participants remaining as motionless as possible (33). The bioelectrical impedance approach has good test–retest reliability (*R* = 0.97 to 0.99).

### Maximal strength

Maximal strength was evaluated using 1-RM for the leg press and chest press, and these measures were also used to estimate training intensity for RT regimens. Before commencing the test, the researchers described the goal, associated risks, discomforts, participant obligations, benefits, questions, and permission. Before the testing session, participants were told to abstain from consuming alcohol for 48 h, caffeinated beverages for 12 h, and meals for 2 h. Water consumption was permitted. Before the test, participants completed a general 10-min warm-up (5 min of slow jogging on a treadmill; 3–5 km/h or elliptical; with 5–10 level) and specialized warm-up exercises (two to three sets of light RT). Participants then completed two attempts, noting their heaviest weight lifted and the number of repetitions. The number of repetitions required to reach fatigue did not surpass ten. The participants were given 3–5 min of rest between trials, and there was no stimulating input throughout testing. Using the formula 1-RM = weight/ (1.0278–0.0278 reps) (34), the maximum strength of participants was estimated after the testing session.

### Muscular power

Upper- and lower-body anaerobic power was assessed via Monark Wingate cycle ergometry (Monark model 894e, Vansbro, Sweden) as previously described (35, 36). Briefly, participants were acquainted with the test and instructed to stay seated in the saddle for the test duration. Participants cycled or cranked against a pre-determined resistance (7.5% of body mass for the lower body test and 5.5% for the upper body test) as fast as possible for 30 s. Participants were verbally encouraged to pedal as hard and fast as possible throughout the whole 30 s test. Peak power output was documented in real-time during the test using Monark Anaerobic test software (3.3.0.0).

### Muscular endurance

After completing the 1-RM and power testing in the morning, participants were instructed to perform leg-and chest press exercises at 75% of the 1-RM to test muscular endurance, denoted as the number of successful repetitions completed before technical failure (i.e., point where participants fail to achieve another repetition in good technical form) in the evening (37).

### Blood tests

Fasting blood samples (5 mL) were taken from the cubital vein using standard procedures following an 8-h overnight fast at the same time of day in pre-and post-testing. Liver enzymes [alanine transaminase (ALT; intra-assay CV: 1.81%; inter-assay CV: 2%)], aspartate aminotransferase (AST; intra-assay CV: 2.01%; inter-assay CV: 2.54%), and gamma-glutamyl transferase (GGT; intra-assay CV: 1.56%; inter-assay CV: 0.92%), creatinine (intra-assay CV: 1.60%; inter-assay CV: 2.24%), and Urea Nitrogen (BUN) (intra-assay CV: 2.20%; inter-assay CV: 3.36%), were measured in serum. Liver and kidney function markers and lipid profiles [low-density lipoprotein (LDL; intra-assay CV: 0.64%; inter-assay CV: 1.37%), high-density lipoprotein (HDL; intra-assay CV: 0.77%; inter-assay CV: 1.80%), cholesterol (intra-assay CV: 1.11%; inter-assay CV: 1.18%)].

were measured in duplicate using Pars Azmoon kits and the spectrophotometric method (DiaSys Diagnostic Systems GmbH, Germany). These were performed after 48 h of rest.

### Resistance training

Before the beginning of the study, all participants performed 1 week of RT, consisting of three exercise sessions, for familiarization before the main training intervention. This phase allowed instruction relating to correct lifting technique, and familiarization with all exercises and equipment, and ensured that the participants initiated the study with a comparable training base (38). Following the preparatory phase, RT began at 50% of 1-RM at the beginning of the training intervention and progressed to 80% of 1-RM in the last week of training (Table 1). Repetitions varied from 6 to 12 and exercises (in order) included leg extension, leg curl, leg press, calf raises, chest press, lat pulldown, lateral raise, biceps curl, triceps extension, abdominal crunch, and back extension. Participant supervision was performed using one personal trainer for five participants (eight personal trainers in total). Maximum strength testing (1-RM) was been performed every 2 weeks to subsequently adjust training intensity. The periodized RT protocols were adapted from previous literature on older adults (9, 39–42). RT volume was calculated using the following formula in each session and was reported weekly (43):

**Table 1:** Resistance training protocol.

Week	Session / wk	Set	Rest (sec)	Repetition	Repetition cadence	Load (% 1-RM)
1	3	3	30	12	1–2 s concentric 2 s eccentric	50
2	3	3	30	12		50
3	3	3	45	10		60
4	3	3	45	10		60
5	3	4	60	8		70
6	3	4	60	8		70
7	3	4	75	6		80
8	3	4	75	6		80

Estimated training volume = [repetitions (*n*) × sets (*n*) × load or selected weight (kg)].

### Diet

Participants completed six 24 h dietary logs (4 non-consecutive weekdays and 2 non-consecutive weekend days) to determine habitual protein intakes. To assist in achieving their targeted protein intake (i.e., 0.8 or 1.6 g/kg/d), participants consumed 0.4 g/kg of dietary protein after each session and other meals based on the recommendations for older adults to maximally stimulate muscle protein synthesis (7). Participants attended consultations with an accredited practicing dietitian every week, where they were provided guidelines to reach protein and energy needs. Protein quantities were consumed *via* foods, and habitual dietary protein intake remained stable throughout the intervention for both groups. Carbohydrate and fat intake were suggested to be within the Acceptable Macronutrient Distribution Range for these macronutrients (45–65% and 20–35% TEI for carbohydrate and fat, respectively). Participants were asked to remain in a positive energy balance to alleviate any potential of energetic stress-related interferences to anabolic adaptations (44, 45). Food records were kept daily by participants throughout the study using “My Fitness Pal” mobile phone application (MyFitnessPal Inc., United States). All dietary intake data were analyzed using (Diet Analysis Plus, version 10; Cengage) to ensure the same food database was used for all analyses.

### Statistical analysis

*A priori* sample size calculation was conducted using G-power 3.1.9.2 software. The rationale for the sample size was based on our previous work which documented significant improvements in lean mass after 8 weeks of high protein diet in older males (8). By utilizing the equation for effect size (ES) [(mean before-mean after the high protein diet)/the pooled standard deviation], this study revealed an ES of 0.34 [(43.9–45.2)/3.78]. In the present study and based on *α* = 0.05, a power (1-β) of 0.80, and an ES = 0.34 (highest approximate effect size), a total sample size of at least 34 participants (*n* = 17 per group) was needed for sufficient power to detect significant changes in the primary outcome of lean mass. We recruited 20% more participants due to potential dropouts. The normality of the distribution of all variables was evaluated before performing statistical analyzes using the Shapiro–Wilk test; there were no missing values at any time point. Baseline characteristics (at PRE) between groups were reported using mean (SD). The student’s t-test was used for group comparisons at baseline. Effects of training and nutritional interventions on dependent variables were analyzed using analysis of covariance (ANCOVA) to determine the differences between the groups over time. Training volume was analyzed using repeated measures of ANOVA. Pearson’s simple linear regressions were performed with a 95% confidence interval (CI). Values between 0 and 0.3 (0 and-0.3) indicate a weak positive (negative) linear relationship through a shaky linear rule. Values between 0.3 and 0.7 (−0.3 and − 0.7) indicate a moderate positive (negative). Values between 0.7 and 1.0 (−0.7 and − 1.0) indicate a strong positive (negative) (46). All analyses were performed using SPSS 26, and figure production was performed using GraphPad Prism (version 8.4.3) and adobe illustrator artwork (version 25).

## Results

### Participant characteristics

**Figure 2:**
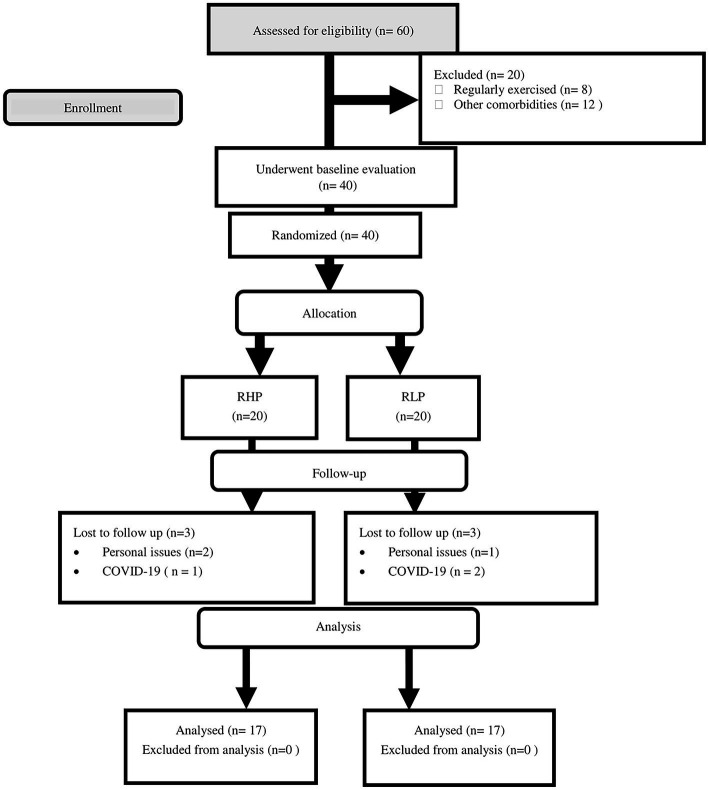
Flow chart of the study design.

Sixty participants were assessed for eligibility and twenty did not meet the inclusion criteria (Figure 2). Three participants from each group (personal issues and COVID-19) withdrew from the study. There were no significant between-group differences in all baseline characteristics (Table 2). There were no differences between groups for PSQI (*p* = 0.556) and GHQ-28 (*p* = 0.095).

**Table 2:** Baseline characteristics of the participants [mean (SD)].

	RHP	RLP
Measure		
Anthropometry, body composition, health, and sleep questionnaires		
Age (y)	61 (2)	62 (3)
Stature (cm)	176.6 (3.6)	175.8 (3.7)
Body mass (kg)	82.1 (5.2)	81.8 (5.6)
BMI (kg.m^−2^)	23.2 (1.2)	23.2 (1.4)
SMM (kg)	33.7 (2.2)	33 (2.3)
BFP (%)	27.1 (2.3)	27.3 (1.5)
PSQI	2.8 (1.2)	3.2 (1)
GHQ-28	21.2 (5.3)	21.8 (8.8)
Performance		
Chest press strength (kg)	40.1 (2.7)	41.8 (3.8)
Chest press endurance (r)	12.2 (2.2)	11.8 (2.4)
Leg press strength (kg)	65.8 (4.1)	66.4 (4.6)
Leg press endurance (r)	14.4 (2.9)	15.3 (3.1)
Upper body power (w)	300.9 (18.6)	295.4 (18.4)
Lower body power (w)	444.1 (15.7)	441.4 (17.5)
Biochemical markers		
GGT (u/L)	20.9 (5)	22.7 (5.8)
AST (u/L)	24.1 (6.9)	21.2 (6.5)
ALT (u/L)	21.7 (6.4)	22.5 (6.3)
Urea (mg/dL)	17.1 (5.1)	18.1 (3.4)
Creatinine (mg/dL)	0.80 (0.17)	0.84 (0.20)
HDL (mg/dL)	53.8 (10)	49.5 (7.9)
LDL (mg/dL)	95 (12.7)	93.2 (9.7)
Cholesterol (mg/dL)	158 (19.9)	156.8 (20.1)

Values are presented as mean ± standard deviation. Abbreviations: PSQI, Pittsburgh Sleep Quality Index; GHQ-28, General Health Questionnaire; BMI, body mass index; SMM, skeletal muscle mass; BFP, body fat percentage; GGT, gamma-glutamyl transferase; AST, aspartate aminotransferase; ALT, alanine aminotransferase; HDL, high-density lipoprotein; LDL, low-density lipoprotein; y, year; cm, centimeter; kg, kilogram; kg.m^−2^, kilogram.meter^−2^; g, gram; %, percentage; r, repetition; w, watt; pg./ml, picograms/milliliter; ng/ml, nanogram/milliliter; u/L, unit/liter; mg/dL, milligrams*/*deciliter; RHP, RT + 1.6 g/kg/d; RLP, RT + 0.8 g/kg/d.

### Body composition

**Figure 3:**
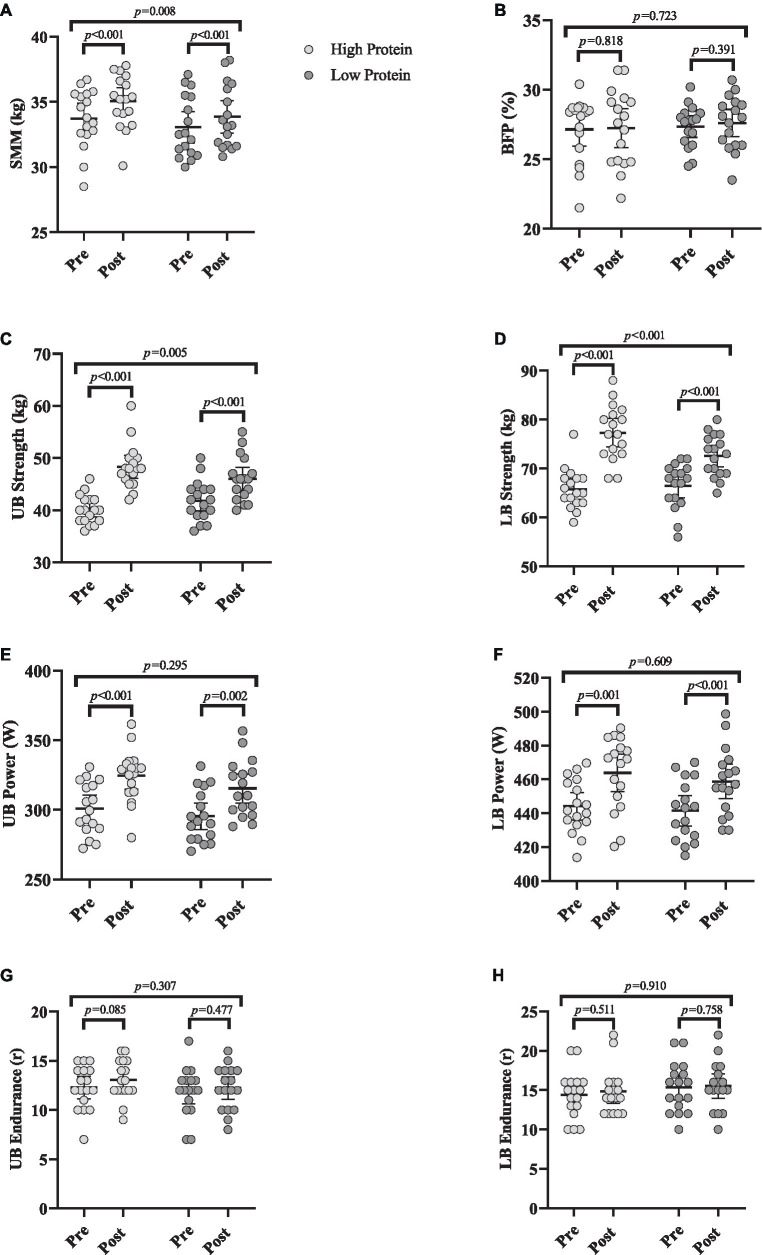
Effects of resistance training in combination with high and low protein diet on body composition and muscular performance. **(A)** Skeletal muscle mass (SMM; kg), **(B)** body fat percentage (BFP; %), **(C)** upper body strength (UB strength; kg), **(D)** lower body strength (LB strength; kg), **(E)** upper body power (UB power; w), **(F)** lower body power (LB power; w), **(G)** upper body endurance (UB endurance; R), **(H)** lower body endurance (LB endurance; R). *n* = 17 per group, error bars represent 95% confidence interval (CI), *p*-values above pre to post indicate paired sample *t*-test results, and *p*-values above groups indicate between-group differences.

Changes in body composition throughout the intervention are shown in Figure 3. Body mass [RHP = 1.7 kg (95% CI = 1.3 to 2.2, *p* < 0.001) and RLP = 0.9 kg (95% CI = 0.62 to 1.1, *p* < 0.001)], SMM [RHP = 1.3 kg (95% CI = 0.95 to 1.6, *p* < 0.001) and RLP = 0.7 kg (95% CI = 0.5 to 1, *p* < 0.001), Figure 3A] all significantly increased from baseline to post-intervention in both groups. However, BFP remained unchanged (Figure 3B) in both groups over time (*p* > 0.05). ANCOVA results indicated significant between-group differences for body mass (*p* = 0.002) and SMM (*p* = 0.008) with greater changes in the RHP group over time.

### Muscular performance

Changes in muscular performance throughout the intervention are shown in Figure 3. Chest press [RHP = 8.2 kg (95% CI = 6.1 to 10.3, *p* < 0.001) and RLP = 4.1 kg (95% CI = 2.7 to 5.5, *p* < 0.001), Figure 3C], leg press strength [RHP = 11.5 kg (95% CI = 9.1 to 13.9, *p* < 0.001) and RLP = 6.1 kg (95% CI = 4.4 to 7.7, p < 0.001), Figure 3D] all significantly increased from baseline to post-intervention in both groups. Upper body [RHP = 23.6 w (95% CI = 14.8 to 32.4, p < 0.001) and RLP = 19.9 w (95% CI = 8.7 to 31.1, *p* = 0.002), Figure 3E] and lower body power [RHP = 19.6 w (95% CI = 9.2 to 30, *p* = 0.001) and RLP = 17.2 w (95% CI = 12.2 to 22.3, *p* < 0.001), Figure 3F] significantly increased from baseline to post-intervention in both groups. However, upper and lower body endurance remained unchanged (Figures 3G,H, respectively) over time (*p* > 0.05). ANCOVA results showed that the gains in the upper (*p* = 0.005) and lower body strength (*p* < 0.001) were significantly greater in RHP over time. However, there were no between-group changes in upper and lower body power (*p* > 0.05).

### Correlations

**Figure 4:**
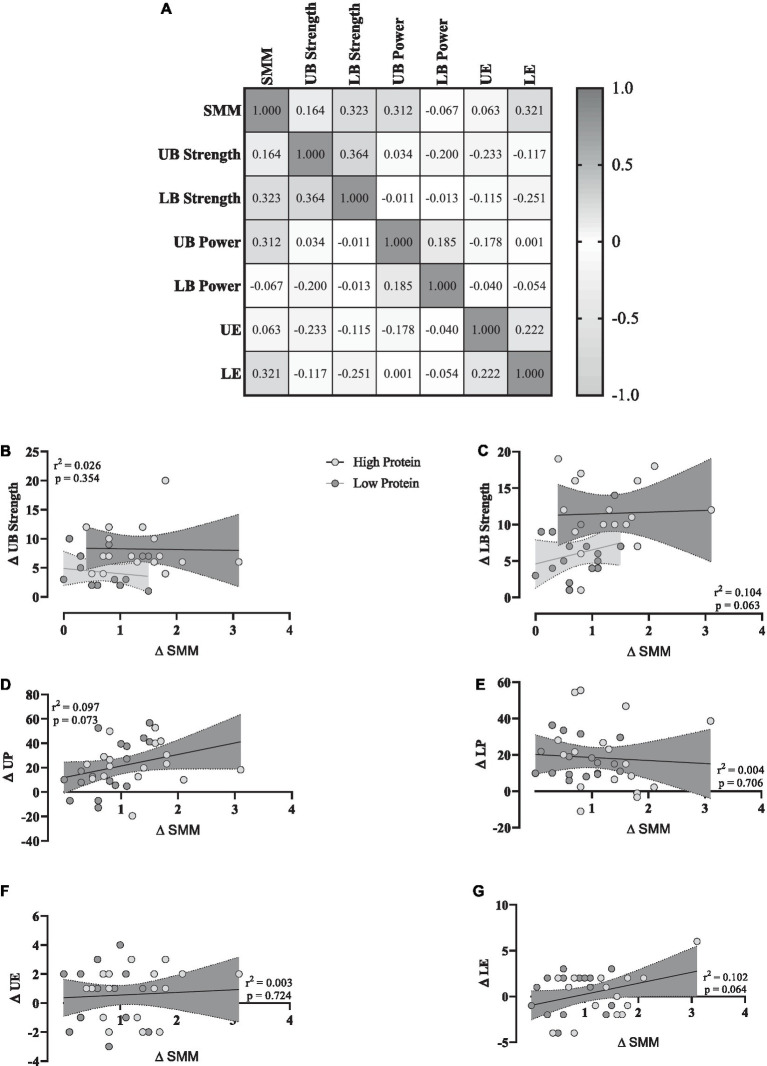
**(A)** Correlation matrix of Δ SMM and performance variables, *r* values as shown. The key indicates the magnitude of *r* (grey = −1 or 1, white = 0). **(B–G)** linear regression (Pearson’s) of Δ (performance) as a function of Δ SMM (kg). Linear regression is indicated by a solid red line, 95% confidence intervals are indicated by cloud and grey zones.

To investigate any potential relationships between training-induced changes in SMM (Δ SMM) and changes in muscular performance (Δ performance variable, independently of RHP or RLP group), a correlation matrix was generated (Figure 4A). Lower body strength (Figure 4C), upper body power (Figure 4D), and lower body endurance (Figure 4G) showed moderate positive relationships with Δ SMM, while upper body strength (Figure 4B) and upper body endurance (Figure 4F) showed a weak positive relationship. However, lower body power (Figure 4E) showed a weak negative relationship. For linear regression of individual Δ (performance variable) as a function of Δ SMM, data were examined by the extra sum-of-squares F test to first consider if pooled data could be considered as a single model. All data except for Δ upper and lower body strength were considered a single group. All data showed a non-significant relationship with changes in SMM.

### Biochemical markers

**Figure 5:**
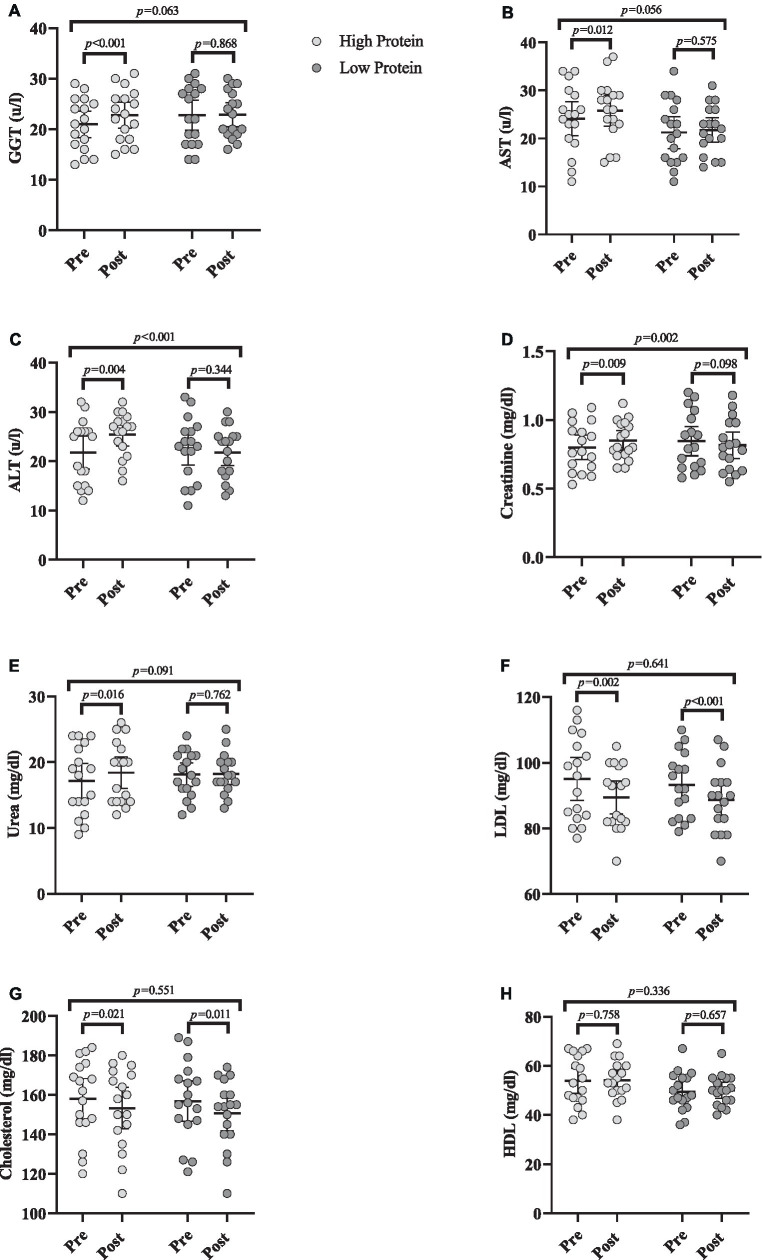
Effects of resistance training in combination with high and low protein diet on biochemical markers. **(A)** gamma-glutamyl transferase (GGT [(u/L)]), **(B)** Aspartate transaminase (AST [(u/L)]), **(C)** Alanine transaminase (ALT [(u/L)]), **(D)** Creatinine (mg/dL), **(E)** Urea (mg/dL), **(F)** low-density lipoprotein (LDL [mg/dL]), **(G)** Cholesterol (mg/dL), **(H)** High-density lipoprotein (HDL [mg/dL]). *n* = 17 per group, error bars represent 95% confidence interval (CI), *p*-values above pre to post indicate paired sample *t*-test results, and *p*-values above groups indicate between-group differences.

Changes in biochemical markers throughout the intervention are shown in Figure 5.

#### Liver function

GGT [RHP = 1.8 (u/L) (95% CI = 1.1 to 2.5, *p* < 0.001), Figure 5A], AST [RHP = 1.7 (u/L) (95% CI = 0.4 to 2.9, *p* = 0.012), Figure 5B], and ALT [RHP = 3.6 (u/L) (95% CI = 1.3 to 5.9, *p* = 0.004), Figure 5C] significantly increased from baseline to post-intervention in RHP group. ANCOVA results indicated that there was only a significant between-group difference for ALT being greater in RHP compared to RLP (*p* < 0.001).

#### Kidney function

Creatinine [RHP = 0.05 mg/dL (95% CI = 0.01 to 0.08, *p* = 0.009), Figure 5D] and urea [RHP = 1.2 mg/dL (95% CI = 0.2 to 2.2, *p* = 0.016), Figure 5E] significantly increased from baseline to post-intervention in RHP group. ANCOVA results indicated that the increase in creatinine in RHP was significantly greater than in RLP (*p* = 0.002).

#### Lipid profile

LDL [RHP = −5.5 mg/dL (95% CI = −8.7 to −2.4, *p* = 0.002) and RLP = −4.5 mg/dL (95% CI = −5.9 to −3, *p* < 0.001), Figure 5F] and cholesterol [RHP = −4.7 mg/dL (95% CI = −8.5 to −0.8, *p* = 0.021) and RLP = −6.1 mg/dL (95% CI = −10.7 to −1.6, *p* = 0.011), Figure 5G] significantly decreased from baseline to post-intervention in both groups. However, HDL remained unchanged (Figure 5H) over time (*p* > 0.05). ANCOVA indicated no significant between-group difference for any marker (*p* > 0.05).

#### Dietary assessments

No adverse events were reported from both groups. Average dietary intakes at baseline and throughout the intervention are presented in Table 3. There was no significant difference between groups at baseline for any average daily nutrient and energy intake (*p* > 0.05). Protein intake [RHP = 0.8 g/kg/d (95% CI = 1 to 0.6, *p* < 0.001)] significantly increased in RHP from pre-to post-intervention. However, no changes in carbohydrates, fat, and energy intakes were observed (*p* > 0.05).

**Table 3:** Average relative dietary intake at baseline and throughout the 8-week training intervention.

	Time	
	Baseline	Training
Energy (kcal/kg/d)		
RHP	26.2 ± 5.3	27.6 ± 4.5
RLP	28.4 ± 4.6	27.3 ± 4
Protein (g/kg/d)		
RHP	0.8 ± 0.3	1.6 ± 0.02 ^a^
RLP	0.7 ± 0.1	0.8 ± 0.1
Carbohydrate (g/kg/d)		
RHP	3.9 ± 1	3.7 ± 0.9
RLP	4.7 ± 1.2	4.4 ± 0.9
Fat (g/kg/d)		
RHP	0.8 ± 0.2	0.6 ± 0.2
RLP	0.7 ± 0.2	0.6 ± 0.1

a *p* < 0.05 different from baseline.

RHP, RT + 1.6 g/kg/d; RLP, RT + 0.8 g/kg/d.

#### Training volume

**Figure 6:**
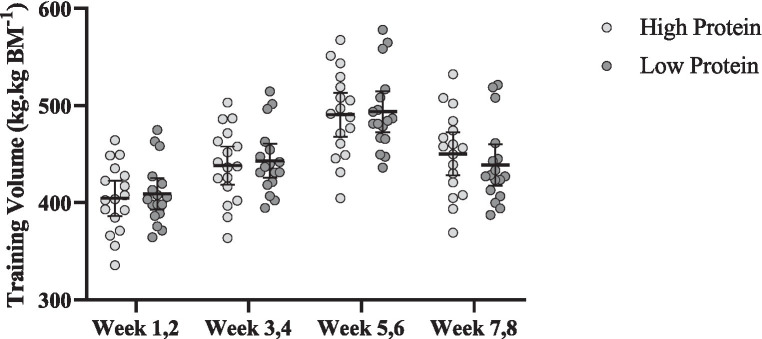
Effects of resistance training in combination with high and low protein diet on relative estimated training volume. *n* = 17 per group, and error bars represent 95% confidence interval (CI).

Changes in training volume throughout the intervention are shown in Figure 6. There was a significant main effect of time for training volume (*p* < 0.001). Training volume [RHP = 34 kg.kg BM^−1^ (95% CI = 38.8 to 29.2, *p* < 0.001) and RLP = −29.9 kg.kg BM^−1^ (95% CI = 35.6 to 24.1, *p* < 0.001), Figure 6] significantly increased from pre to post in both groups. However, no significant interaction or group effect was noted (*p* > 0.05).

## Discussion

The objective of the present study was to compare the effects of 8 weeks of RT in the combination of 1.6 g/kg/d of protein vs. 0.8 g/kg/d on body composition, muscular performance (strength, power, and endurance), and biochemical markers of liver and kidney function in a cohort of healthy, untrained older ex-military males. Our results demonstrate greater gains in SMM and muscular strength (both upper and lower body) with a daily protein intake of 1.6 g/kg/d (RHP group) compared to participants ingesting 0.8 g/kg/d (RLP group) in ex-military older adults concomitant with 8-weeks of RT. These findings are of clinical importance since such improvements in SMM and muscular strength are important to reducing the adverse effects of sarcopenia on musculoskeletal function and health.

### Body compositional and muscular adaptations

Beginning at 50 years of age, the steady reduction in strength and SMM becomes noticeable (47), with SMM and strength/power declining at rates of 0.8–1% and 2–3% each year, respectively (2). Maintenance of skeletal muscle strength is an important factor to preserve functional capacity and independent living with advancing age (47). Nevertheless, even in very physically active older adults (e.g., training four to five or more sessions per week), there have been noted deteriorations in leg strength of 3–5% *per annum* (48). The greater increase in SMM and strength from a high-protein diet in our current work supports previous findings in older males. For instance, 12 weeks of supervised (3 sessions/wk) RT in combination with a high protein diet (1.8 g/kg/d) in older (~63 years) males (*n* = 6) and females (*n* = 3) already consuming 1.2 g/kg/d resulted in a 0.6% increase in fat-free mass (FFM), absolute and relative (53–78%) maximal 1-RM strength (leg press, chest press and lat pulldown) compared to baseline. However, the gains in fat-free mass and absolute 1-RM strength were not statistically significant compared to the control group (1.4 g/kg/d). In regards to power, no change was noted in the countermovement jump. Overall, these results suggest that the intake of a high-protein dairy milk beverage, in combination with RT, elicits greater effects on skeletal muscle strength, but not muscle hypertrophy or power outcome, than consuming the dairy milk beverage or RT in isolation (49). When compared to the results noted in the present study, it seems that the main influencer of the lack of difference in fat-free mass between a high protein diet in combination with RT in comparison to RT in isolation and control is the dosage of protein ingestion per day, which was not statistically different. However, in the present study, the dose of protein in the high-protein diet group (1.6 g/kg/d) was two times higher than the low-protein diet (0.8 g/kg/d). Previous studies have shown that older adults’ maximum 1-RM leg strength improves by >25% following 12 weeks of RT (50, 51) which is consistent with our findings. In line with our results, a recent meta-analysis demonstrated that protein supplementation (20 ± 18 g/d) increases leg press 1-RM (33%) in older adults (mean age: 62 ± 6 y) (52), which challenges earlier studies with exercise interventions that have not witnessed protein supplementation to further gains in maximal leg press 1-RM during RT in healthy community-dwelling and active older adults in comparison to placebo or training in isolation group (50, 51). The difference in the average age between the participants in the present investigation (61 ± 2 years) and those in the prior literature (≥70 years) is one probable explanation for the favorable outcomes in the present. Second, the quantity of daily protein consumed by the supplementation groups may not have been adequate to produce a meaningful difference in strength across groups.

For instance, cohorts receiving additional milk servings were ingesting 1.3–1.4 g/kg/d of protein at pre-intervention (50, 51). Even though this is greater than the recommendations for older adults (≥1.2 g/kg/d) to combat sarcopenia, it is lower (1.6 g/kg/d) than the threshold suggested to support significant changes in muscle hypertrophy and strength during prolonged RT in healthy active adults (7, 53–55). Muscle hypertrophy and strength adaptations may necessitate a higher protein intake (e.g., ≥ 1.6 g/kg/d) than what is presently suggested for active older individuals (≥1.2 g/kg/d) in light of the findings of this study. Due to the importance of dietary distribution during the day in older adults (7), particularly as research suggests that the distribution of protein is often inadequate at breakfast, lunch, and post-exercise in older adults (56), we instructed our participants to consume 0.4 g/kg at each meal to maximally stimulate muscle protein synthesis (7). This may be another important factor in increases of SMM and strength.

In contrast to changes in SMM and strength, no between-group differences were observed in the magnitude of increases in muscular power (both upper and lower body), which is in agreement with a recent study in which countermovement jump was measured as muscular power (49). Also, in the present study, there was no relationship between SMM and muscular strength with power outcomes. These results indicate that the gains in muscular power are independent of protein ingestion or SMM values and may be more dependent on exercise training mode. In support, previous work suggests high-velocity/power RT, defined by completing the concentric phase of each movement as quickly as feasible while the eccentric phase is executed slowly and under control (57), significantly moderated the influences of RT on muscle power compared to conventional RT, indicating that high-velocity RT is superior to traditional RT for muscular power gains in older adults (58, 59). As muscular power is the product of contraction force and movement velocity, it is conceivable, biomechanically, that fast concentric contractions would improve muscle power more than traditional RT. Since we used traditional RT in the present study, the lack of difference between groups is perhaps unsurprising. Thus, while a high-protein diet may not be optimal for maximizing muscle power adaptations in older individuals, performing high-velocity/power RT in such cohorts should be considered with caution as such movements may increase the likelihood of losing balance or muscle tears due to the rapid nature of muscle contraction in such movements.

### Biochemical markers

High protein diets have been linked to possible negative effects on renal function, namely glomerular filtration rate (22–24). Specifically, it has been proposed that high and persistent consumption of dietary protein may eventually lead to glomerular damage, renal impairment, and kidney failure (21). Although such associations may be more probable in those with impaired renal function (such as chronic kidney disease) (60), the relationship between increased dietary protein availability and impaired kidney and liver function seems to be significantly less pronounced in individuals with healthy kidney function (61). Considering the protein component of our high dietary protein intervention group, which was two-fold higher than the most current national recommended daily allowance for daily protein intake, we evaluated several biochemical markers of lipid, kidney, and liver function. Our results indicated within-group increases in GGT, AST, ALT, creatinine, and urea while only ALT and creatinine values were greater in RHP compared to the RLP group. Compared to referenced ‘normal’ ranges, post-intervention levels of GGT, ALT, AST, urea and creatinine were all within normal reference ranges in the RHP group (62–65). It cannot be discounted that increased levels of these markers outside ‘normal’ healthy ranges may occur over longer periods (i.e., months to years) with a continued high protein diet and possible deterioration in kidney function with advancing age. Nonetheless, our data show that older adults with healthy kidney function can consume a high protein diet (i.e., 1.6 g/kg/d) over an 8-week period without an adverse effect on liver or kidney function. Finally, both groups similarly decreased LDL and cholesterol with no changes in HDL, indicating the role of RT independent of protein ingestion on blood lipid health.

There were several limitations of our present work. Firstly, bioelectrical impedance was used to assess body composition which is not as accurate as dual-energy x-ray absorptiometry (a more suitable method for body composition measurements) although is still a reliable method (66, 67). Additionally, we lacked control over age-matched individuals that were not previously in the military. Regarding strengths of our study design, we investigated a cohort of participants that are under-represented in the skeletal muscle and nutrition research field. Moreover, we used nutrition software to track macronutrient intake which is rarely used in the literature, in older adults.

In conclusion, a daily intake of 1.6 g/kg/d protein was superior to 0.8 g/kg/d (current recommended daily intake) for promoting greater improvements in SMM and muscle strength and thus may be a more suitable level of intake for promoting such adaptive responses. Notwithstanding observed between-group differences in ALT and creatinine and the fact that levels remained within normal ranges, it is feasible to conclude that this daily protein intake is efficacious and well tolerated by healthy, untrained older ex-military males. Such knowledge is of critical importance for older populations since gains in SMM and strength can reduce the many deleterious effects of sarcopenia, reduce the risk of falls, and improve the quality of independent living (7, 68). Therefore, older adults could use the intake of 1.6 g/kg/d of protein to improve muscular (e.g., muscle strength) and body composition (e.g., SMM).

## Data availability statement

The raw data supporting the conclusions of this article will be made available by the authors, without undue reservation.

## Ethics statement

The studies involving human participants were reviewed and approved by the Baqiyatallah University of Medical Sciences. The patients/participants provided their written informed consent to participate in this study.

## Author contributions

RB and AS: conceptualization and project administration. RB: methodology, writing—original draft preparation, formal analysis, and investigation. RB and EN: software. RB and VS: validation. AS: resources, supervision, and funding acquisition. RB, HG, and HF: data curation. DC, RB, and FD: writing—review and editing. All authors have read and agreed to the published version of the manuscript.

## Funding

This research was supported by the Baqiyatallah University of Medical Sciences, Tehran, Iran.

## Conflict of interest

The authors declare that the research was conducted in the absence of any commercial or financial relationships that could be construed as a potential conflict of interest.

## Publisher’s note

All claims expressed in this article are solely those of the authors and do not necessarily represent those of their affiliated organizations, or those of the publisher, the editors and the reviewers. Any product that may be evaluated in this article, or claim that may be made by its manufacturer, is not guaranteed or endorsed by the publisher.

## Footnotes

^1^Available at https://www.randomizer.org/.
